# Association of the Genetic Variation in the Long Non-Coding RNA FENDRR with the Risk of Developing Hypertrophic Cardiomyopathy

**DOI:** 10.3390/life12060818

**Published:** 2022-05-30

**Authors:** Elías Cuesta-Llavona, Rebeca Lorca, Valeria Rolle, Belén Alonso, Sara Iglesias, Julian Rodríguez-Reguero, Israel David Duarte-Herrera, Sergio Pérez-Oliveira, Alejandro Junco-Vicente, Claudia García Lago, Eliecer Coto, Juan Gómez

**Affiliations:** 1 Hospital Universitario Central de Asturias (HUCA), 33011 Oviedo, Spain; UO218576@uniovi.es (E.C.-L.); rebeca.lorca@sespa.es (R.L.); belen.montanel@hotmail.com (B.A.); saraiglesiasalvarez@gmail.com (S.I.); josejulian.rodriguez@sespa.es (J.R.-R.); israelddh@hotmail.com (I.D.D.-H.); UO258050@uniovi.es (S.P.-O.); alejandro.junco@sespa.es (A.J.-V.); 2 Instituto de Investigación Sanitaria del Principado de Asturias (ISPA), 33011 Oviedo, Spain; Avleria77@gmail.com (V.R.); claudiagarcilago@gmail.com (C.G.L.); 3 Redes de Investigación Cooperativa Orientadas a Resultadosen Salud (RICORs), 28029 Madrid, Spain; 4 Unidad de Cardiopatías Familiares del HUCA, 33011 Oviedo, Spain; 5 Medicicine Department, Universidad de Oviedo, 33003 Oviedo, Spain; 6 CIBER-Enfermedades Respiratorias, 28029 Madrid, Spain

**Keywords:** lncRNAs, Hypertrophic Cardiomyopathy, NGS

## Abstract

**Background:** In around 40–60% of Hypertrophic Cardiomyopathy (HCM) cases pathogenic variants are not identified. Our aim was to evaluate the possible association of lncRNAs with the risk of developing HCM. **Methods:** We sequenced 10 lncRNAs coding genes that have been associated with cardiovascular disease in a discovery cohort (238 HCM patients and 212 controls) by NGS, and genotyped rs74035787 G>A and rs1424019 A>G polymorphism in a validation cohort (962 HCM patients and 923 controls). Finally, we sequenced the *FENDRR* promoter by Sanger sequencing. **Results:** We observed by NGS that *FENDRR* rs39527, rs39529 and rs40384 polymorphisms were significantly associated with HCM in our cohort (*p* = 0.0284; OR: 0.24, 95%CI: 0.07–0.86). NGS results were confirmed by genotyping rs74035787 polymorphism (*p* = 0.001; OR:0.38, 95%CI: 0.21–0.66). Moreover, it is also associated when stratification by sex (*p* = 0.003; OR:0.20, 95%CI: 0.06–0.53), and age (≥50 years old *p* = 0.001, OR:0.33, 95%CI: 0.16–0.63) Moreover, the risk of HCM in the carriers of the GG genotype of the rs1424019 polymorphism was significantly higher than that of the AA/AG genotypes carriers in the elderly subjects (*p* = 0.045, OR:1.24, 95%CI: 1.01–1.53). On the other hand, we observed significant differences in the rs74035787 A/rs1424019 G haplotype frequency (*p* = 0.0035; OR: 0.20, 95%CI: 0.07–0.59). **Conclusions:** Our study suggested a significant association between *FENDRR* gene variants and HCM.

## 1. Introduction

Hypertrophic Cardiomyopathy (HCM) is the most common hereditary heart disease, with a prevalence of 1/500 (0.2%) [1] and it is caused by pathogenic variants, mainly in genes encoding sarcomere proteins [2,3]. Meanwhile, next generation sequencing (NGS) has revolutionized the study of the genetic basis of heritable diseases andexome sequencing has proven to be an extremely useful tool for identifying new genes associated with various pathologies; however, in around 40–60% of HCM cases, the causal pathogenic variant is not identified, including cases with a family history of the disease. Most pathogenic variants are associated with clinical heterogeneity, incomplete penetrance, and variable phenotypic expressiveness, including carriers in the same family [4]. Studies suggest that there are other genetic factors that could partially explain the risk of developing HCM or modify the phenotype among carriers anda group of candidate genes to be evaluated are those that encode long-non-coding RNAs (lncRNAs), whose variation escapes the informatics prediction tools applicable to the exome. These RNAs are molecules with a length of more than 200 nucleotides which have a role in the modulation of gene expression and signaling pathways. They perform important roles in various physiological processes through epigenetic modulations (gene silencing), regulating alternative splicing or as molecular sponges. In addition, they can interfere with the translation of proteins by blocking, stabilizing or destabilizing the mRNA. In addition, lncRNAs have low levels of expression, conservation, and high tissue specificity. These RNAs are classified by their gene location in sense, intronic sense, antisense, bidirectional, or intergenic lncRNA [5,6]. Furthermore, the expression of a large number of lncRNAs has been characterized in the cardiovascular system both in physiological and pathological conditions (Table 1) [7,8,9,10,11,12,13,14,15,16,17,18].

Regarding HCM, there are studies that have pointed to an association between lncRNAs and this pathology. It was reported that *H19* regulates cardiac hypertrophy through *miR-675* and genetic variation at this lncRNA is associated with the risk of HCM [12,19], that *MIAT* perform like a *miR-150* molecular sponge [13], *MHRT* antagonizes the role of *Brg1* in cardiac hypertrophy [9], *PVT1* modulates the pathological cardiac hypertrophy via miR-196b [18] and *TINCR* regulates cardiac hypertrophy through the epigenetic silencing of *CaMKII* [16];however, only a limited number of lncRNAs have been identified as regulators of cardiac hypertrophy. The aim of this study is to characterize the genetic variations of several lncRNA and their possible role in the risk of developing hypertrophic cardiomyopathy.

## 2. Materials and Methods

### 2.1. Patients and Controls

The study included a discovery cohort of 238 index cases of HCM and 212 healthy controls. All the study participants were Caucasian from the region of Asturias (Northern Spain). The patients were recruited through the Familial Cardiomyopathies Unit of Hospital Universitario Central Asturias, the national reference center for these pathologies. Healthy controls were recruited through the primary care centers of the region. They were an elderly cohort with neither a HCM diagnosis nor alterations in their electrocardiograms. Since HCM is an age-dependent disease, the controls were thus selected to have a mean age higher than the patients to reduce the possibility of not identifying late-onset HCM-candidate variants. An additional validation cohort was recruited to confirm the NGS results. This cohort included 962 HCM patients (199 harboring pathogenic/likely pathogenic variants according to the American College of Medical Genetics and Genomics criteria [20], 20.7%) recruited as the discovery cohort, and 923 healthy controls of the same region. The only inclusion criterion was the informed consent for the genetic study. The main demographic values were obtained from the clinical history. The study was approved by the Ethics Committee of Principado de Asturias (Oviedo, Spain) and informed consent was obtained from all patients or their representatives. Table 2 summarizes the main characteristic of these cohorts.

### 2.2. Next Generation Sequencing

We obtained DNA from blood leukocytes of both cohorts and performed a NGS of a total of 10 lncRNAs genes that have been associated with the cardiovascular system after an exhaustive bibliography review (*H19, KCNQ1OT1, MHRT, CARMEN, FENDRR, TINCR, ANRIL, MIAT, PVT1, MALAT1*) (Supplementary Table S1). NGS were performed in the discovery cohort by semiconductor chips technology (Ion Torrent) in an Ion GeneStudio S5 Plus Sequencer (Thermo Fisher Scientific, Waltham, MA, USA). The detailed procedure was previously reported [2,21]. The raw data was processed with the Torrent Suite v5 software. Reads assembling and variant identification were performed with the Variant Caller (VC). The Ion Reporter (Thermo Fisher Scientific), and HD Genome One (DREAMgenics S.L., Oviedo, Asturias, Spain) software were used for variant annotation, including population, functional, disease-related and in silico predictive algorithms. The Integrative Genome Viewer (IGV, Broad Institute, Cambridge, MA, USA) was used for the analysis of depth coverage, sequence quality, and variant identification.

### 2.3. Genotyping Studies

PCR-RFLP with restriction enzyme *NcoI* was performed in the validation cohort to genotype *FENDRR* rs74035787 G>A polymorphism. The results were visualized in a 4% agarose gel electrophoresis (Supplementary Figure S1). Furthermore, the rs1424019 A>G polymorphism was genotyped by real-time PCR with Taqman probes (Thermo Fisher Scientific, assay id. C_12107263_10: GGGTGAGTGACTGAGCACGCCGCTT[A/G]TTCAGGGGCCTGTGCGGTCTGGGTC).

### 2.4. FENDRR Promoter Sanger Sequencing

Additionally, we sequenced the *FENDRR* promoter in the HCM patients. The main objective was to search for polymorphisms that could be associated with HCM, to define the LD between rs1424019 and other promoter SNPs and identify how these polymorphisms affect the *FENDRR* expression. A fragment that covered the *FENDRR* promoter was amplified with the primers 5′GACCGCCTACCCACTCTC and 5′GCACGCGGAATTCTCTATTATTAT (706 bp). This amplified fragment was Sanger sequenced with BigDye chemistry in an ABI3130xl (Thermo Fisher Scientific, Waltham, MA, USA).

### 2.5. Statistic Analysis

Statistical analyses were performed using R statistical software (version 4.2.0). Continuous variables were expressed as the median [Q1, Q3] and compared with a Mann–Whitney U test. Categorical variables were expressed as a number and percentage n (%) and compared with Fisher’s exact test. Univariate and multivariate logistic regression was used to compare the genotype and allelic frequencies in the polymorphisms identified by NGS and genotyping studies. Regressions were adjusted by sex but not age, as the controls were purposefully older than the cases. In some cases where regular logistic regression could not be performed due to a zero cell, Firth’s bias-reduced penalized-likelihood logistic regression was used. Odds Ratio (OR), 95% confidence intervals (95%CI) and *p*-values are reported. The correlation between the *FENDRR* polymorphisms and the risk of HCM was determined based on the distribution of allele frequency. A value of *p* < 0.05 was considered to be significant. In addition, we performed an online bioinformatic prediction of the effect of the nucleotide changes on RNA folding with the RNA Web Server tool (https://rth.dk/resources/rnasnp/, accesed on 20 March 2022) [22].

## 3. Results

### 3.1. Next Generation Sequencing in Discovery Cohort

**Figure 1:**
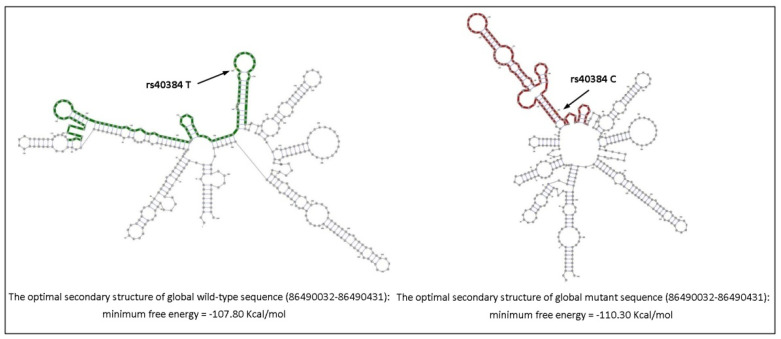
Structural differences in lncRNA *FENDRR* related to the rs40384 T>C polymorphism. The structural folding of *FENDRR* was predicted with http://rth.dk/resources/rnasnp/. Arrows indicate the T/C nucleotide change.

We observed by NGS that *FENDRR* rs39527 A>G, rs39529 G>C and rs40384 T>C polymorphisms (Supplementary Figure S2) were associated with the risk of developing HCM in our cohort by significant differences between the patients and healthy controls (0.006 vs. 0.03; *p* = 0.0284, OR: 0.25, 95%CI: 0.07–0.86). Therefore, we performed an online bioinformatic prediction of the effect of these nucleotide changes in *FENDRR* folding with the RNA Web Server tool. We observed that the rs40384 T>C polymorphism caused important *FENDRR* secondary structure differences (Figure 1).

### 3.2. Genotyping Studies in Validation Cohort

NGS results were confirmed by PCR-RFLP (Table 3 and Table 4) with restriction enzyme *NcoI*, in the validation cohort through genotyping for the rs74035787 G>A polymorphism (0.009 vs. 0.029; *p* = 0.001, OR: 0.38, 95%CI: 0.21–0.66), which is in linkage disequilibrium (LD) with the three *FENDRR* polymorphisms identified by NGS. These data imply a statistics power of 91.4% for this variant. We also genotyped by Taqman assay the rs1424019 A>G polymorphism, a *FENDRR* promoter SNP which could have a role in the regulation of the expression (Table 3 and Table 4). No significant differences were observed between the patients and controls (0.75 vs. 0.74; *p* = 0.333). The allele frequencies for these polymorphisms in our population were close to the reported among individuals from European ancestry (Eurx 1000 genomes; Supplementary Figure S3).

In addition, we stratified the cohort by age (≥50 years old, and <50) and gender (Table 5). The results showed that the risk of developing HCM in the carriers of the A allele (GA/AA) of the *FENDRR* rs74035787 polymorphism was significantly lower than that of the G allele (GG genotype) carriers in the elderly subjects and females (*p* = 0.001, OR: 0.33, 95%CI: 0.16–0.63; *p* = 0.003, OR: 0.20, 95%CI: 0.06–0.53, respectively; Table 5).

The risk of HCM in the carriers of the GG genotype of the rs1424019 polymorphism was significantly higher than that of the AA/AG genotypes carriers in the elderly subjects (*p* = 0.045, OR:1.24, 95%CI:1.01–1.53), meanwhile a trend was shown in males (*p* = 0.063, OR:1.25, 95%CI: 0.99–1.58), but not in the young, and female subjects (*p*> 0.05; Table 5)

Additionally, we calculated the haplotype frequency between the two *FENDRR* SNPs, rs74035787 and rs1424019, in the patients and healthy controls. Significant differences were seen between the patients and healthy controls in the rs74035787 A/rs1424019 G haplotype frequency (0.002 vs. 0.010; *p* = 0.0035, OR: 0.20, 95%CI: 0.07–0.59; (Table 6)).

### 3.3. FENDRR Promoter Sanger Sequencing

Finally, in order to investigate variants in the *FENDRR* which could modify its expression, we sequenced the *FENDRR* promoter in the HCM patients. We identified several variants, all previously reported, including rs1364225 C>G, rs3812976 A>G and rs3841240 TGTTTG/TG polymorphisms. These SNPs were in high LD with rs1424019 A>G; however, we have not identified any rare or not yet described variant in our cohort.

## 4. Discussion

The *FOXF1Adjacent Noncoding Developmental Regulatory RNA* (*FENDRR*) is a lncRNA that is transcribed bidirectionally with *FOXF1* on the opposite strand. Both genes share a region containing their respective promoters [23]. Supplementary Figure S4 shows thecellular localization and expression for the *FENDRR* gene across all cell lines. This lncRNA performs its role through epigenetic modulations, or as molecular sponges of microRNAs and transcription factors. *FENDRR* is involved in the development and progression of various types of cancer by regulating cell proliferation, migration, invasion, and apoptosis [24,25,26] and plays important roles in cardiac lineage involvement through interaction with Polycomb repressive complex 2 (*PRC2*) and the *TrxG/MLL* complex to regulate the activation of a group of cardiac differentiation genes in cardiac progenitor cells [7,8]. Although *FENDRR* is found to be essential for proper heart and body wall development in mice, there is no clear evidence about the role of *FENDRR* in human cardiovascular diseases. A recent study found that *FENDRR* inhibits hypoxia/reoxygenation-induced cardiomyocyte apoptosis by p53 degradation and this would protect myocardial cells from ischemia/reperfusion injury [27].

According to our results, it was observed that the *FENDRR* rs39527 A>G, rs39529 G>C and rs40384 T>C polymorphisms were significantly associated with the risk of developing HCM in our cohort, having a protective role to develop HCM. Furthermore, these results were confirmed by PCR-RFLP in the validation cohort for the rs74035787 G>A polymorphism, which is in LD with the three polymorphisms identified by NGS. Therefore, this study identified the significant protective effect of the rare allele in the *FENDRR* rs39527 G, rs39529 C, rs40384 C and rs74035787 A polymorphisms in this disease in a Spanish cohort. In addition, we observed that the two RNA transcripts for the rs40384 T>C showed important secondary structure differences. This suggests that these polymorphisms would induce structural rearrangements in the *FENDRR* lncRNA that sequester or expose the binding sites of various molecules or transcription factors involved in the cardiac hypertrophy regulation. Moreover, the secondary structure would play a fundamental role in the functionality of lncRNAs.

Additionally, the rs1424019 A>GGG genotype was more frequent in elderly and male patients in the validation cohort. Furthermore, we identified three polymorphisms (rs1364225, rs3812976 and rs3841240) through *FENDRR* promoter Sanger sequencing that were in high LD with rs1424019. These SNPs could modify *FENDRR* expression in different tissues. Several studies showed by RNAseq that *FENDRR* expression was higher in lung, urinary bladder, gallbladder, esophagus, prostate, and the colon, and was almost undetectable in heart, kidney and liver [28,29]. On the other hand, dysregulation *FENDRR* expression has been linked to tumorigenesis, fibrosis, and inflammatory diseases [23]. In a recent study, it was observed a significant negative correlation of *FENDRR* expression levels with left ventricular mass index in peripheral blood mononuclear cells of patients with essential hypertension in relation to left ventricular hypertrophy, suggesting that *FENDRR* could possibly have a cardioprotective role [30]. However, another study observed that *FENDRR* is required for the pathogenesis of cardiac fibrosis in a mouse heart by sponging the *miR-106b*, which is a negative regulator of pro-fibrotic *Smad3* [31]. In addition, the NIH Genotype-Tissue Expression (GTEx) project showed that the GG genotype in rs1424019 polymorphism had lower *FENDRR* expression than the AA and AG genotype in esophagus muscularis and colon [28]. According to Kontaraki JE. et al. and GTEx Consortium results, our study suggests that the GG genotype (58.4% in patients vs. 55.3% in controls) would decrease the *FENDRR* expression in the heart and increase the risk of developing HCM. Meanwhile the AA + AG genotype was more frequent in controls (41.61% vs. 44.7%), which could increase the *FENDRR* expression and decrease the risk of developing this pathology. Therefore, these promoter polymorphisms would affect the *FENDRR* expression in the heart and the risk of developing HCM. This could be due to the fact that *FENDRR* overexpression inhibits cell proliferation, since this lncRNA performs through an interaction with *PRC2* to promote the target gene promoters’ methylation, thus reducing the cardiac genes expression; however, the functional relevance of these changes requires validation by functional studies.

To further analyze the genetic association between the risk of HCM and *FENDRR* polymorphisms in different subpopulations, we performed stratified analyses to investigate the effects of age and gender. We found that elderly people and female carriers of the rs74035787 A allele (GA/AA) were significantly lower risk than GG carriers. These results are in concordance with the proved fact that females have a lower prevalence of HCM, and a latter onset age [32,33,34,35,36,37,38]. In addition, the risk of HCM in the rs1424019 polymorphism G allele (GG genotype) carriers in the elderly patients was higher than that of the A allele (AA/AG) carriers.

Finally, we found significant differences in the rs74035787 A/rs1424019 G haplotype frequency, with it being lower in patients than in the controls. This suggests that this haplotype reduces the risk of developing HCM and performs a significant protective effect in this pathology. In this way, the A allele in the rs74035787 polymorphism could neutralize the pro-hypertrophic effect of the G allele in the rs1424019 polymorphism.

## 5. Limitations of the Study

Baseline characteristics as body mass index, comorbidities…, etc., were not available either for the patients or the healthy controls. Moreover, functional studies to measure *FENDRR* expression levels had not been performed. These results should be validated by additional studies.

## 6. Conclusions

In conclusion, we identified a significant association between rs39527 G, rs39529 C, rs40384 C and rs74035787 A *FENDRR* alleles in HCM, which suggests a protective role of these alleles, mainly in females. These variants could involve a change in the structure of *FENDRR* that would modify the regulation of gene expression controlled by this lncRNA. To our knowledge, this is the first study which associates *FENDRR* lncRNA with Hypertrophic Cardiomyopathy, increasing the scope of the genetic mechanism involved in the disease; however, the functional relevance of these changes requires additional experimental validation.

## Figures and Tables

**Table 1:** Examples of lncRNAs and their mechanism of action in the cardiovascular system in normal physiological conditions or disease states.

lncRNAs	Mechanism of Action	References
*FENDRR*	Cardiovascular embryogenesis, interaction with PRC2	[7,8]
*MHRT*	Antagonizes the role of Brg1 in cardiac hypertrophy	[9]
*CARMEN*	Regulation of cardiac differentiation	[10]
*MALAT1*	Regulates the proliferation of endothelial cells and cardiomyocytes, epigenetic and alternative splicing regulation	[11]
*H19*	Regulates cardiac hypertrophy through *miR-675*	[12,19]
*MIAT*	Regulates cardiac hypertrophy, *miR-150* molecular sponge	[13]
*KCNQ1OT1*	Long QT syndrome, regulator of gene expression	[15]
*TINCR*	Regulates cardiac hypertrophy, epigenetic silencing of *CaMKII*	[16]
*ANRIL*	Structure and function of vascular smooth muscle, regulator of gene expression	[17]
*PVT1*	Modulates the pathological cardiac hypertrophy via *miR-196b*	[18]

**Table 2:** Main characteristics of the discovery and validation cohort.

	Discovery			Validation		
	Controls	Patients	*p*-Value	Controls	Patients	*p*-Value
	(N = 212)	(N = 238)		(N = 923)	(N = 962)	
**Age**	72.0 [64.0, 76.0]	61.0 [51.0, 69.0]	<0.001	66.0 [55.8, 78.0]	60.0 [48.0, 69.0]	<0.001
**Sex**						
Men	91 (45.0%)	148 (63.0%)	<0.001	518 (56.1%)	620 (64.4%)	<0.001
Women	111 (55.0%)	87 (37.0%)		405 (43.9%)	342 (35.6%)	
**Sarcomere**						
No	-	186 (78.2%)	-	-	763 (79.3%)	-
Yes	-	52 (21.8%)		-	199 (20.7%)	

**Table 3:** Descriptive results of genotype and allele frequencies of the rs74035787 and rs1424019 polymorphisms in the validation cohort. Results are shown as overall cases, stratified by gender, and by age (≥50 years old).

	Patients				Controls				Total Patients	Total Controls
	Men	Women	≥50	< 50	Men	Women	≥50	<50		
	(N = 620)	(N = 342)	(N = 691)	(N = 271)	(N = 518)	(N = 405)	(N = 763)	(N = 160)	(N = 962)	(N = 923)
**rs74035787**										
GG (n (%))	607 (97.9%)	338 (98.8%)	680 (98.4%)	265 (97.8%)	499 (96.3%)	382 (94.3%)	727 (95.3%)	154 (96.3%)	945 (98.2%)	881 (95.4%)
GA (n (%))	13 (2.10%)	4 (1.17%)	11 (1.59%)	6 (2.21%)	19 (3.67%)	23 (5.68%)	36 (4.72%)	6 (3.75%)	17 (1.77%)	42 (4.55%)
G (Freq.)	0.990	0.994	0.992	0.989	0.982	0.972	0.976	0.981	0.991	0.977
A (Freq.)	0.011	0.006	0.008	0.011	0.018	0.028	0.024	0.019	0.009	0.023
**rs1424019**										
AA (n (%))	53 (8.55%)	26 (7.60%)	55 (7.96%)	24 (8.86%)	42 (8.11%)	30 (7.41%)	58 (7.60%)	14 (8.75%)	79 (8.21%)	72 (7.80%)
AG (n (%))	212 (34.2%)	109 (31.9%)	222 (32.1%)	99 (36.5%)	208 (40.2%)	133 (32.8%)	286 (37.5%)	55 (34.4%)	321 (33.4%)	341 (36.9%)
GG (n (%))	355 (57.3%)	207 (60.5%)	414 (59.9%)	148 (54.6%)	268 (51.7%)	242 (59.8%)	419 (54.9%)	91 (56.9%)	562 (58.4%)	510 (55.3%)
A (Freq.)	0.256	0.235	0.240	0.271	0.282	0.238	0.263	0.259	0.249	0.263
G (Freq.)	0.744	0.765	0.760	0.729	0.718	0.762	0.737	0.741	0.751	0.737

**Table 4:** Univariate and multivariate analysis of rs74035787 and rs1424019 variants. Since healthy controls were selected older on purpose, only sex was added to the multivariate analysis.

		Univariate			Multivariate		
	Predictors	Odds Ratio	CI	*p*-Value	Odds Ratio	CI	*p*-Value
**rs74035787**	GG	Reference			Reference		
	GA	0.38	0.21–0.66	0.001	0.38	0.21–0.67	0.001
	Women				0.71	0.59–0.85	<0.001
	G	Reference			Reference		
	A	0.38	0.21–0.66	0.001	0.39	0.21–0.67	0.001
	Women				0.71	0.62–0.81	<0.001
**rs1424019**	GG	Reference			Reference		
	AA o AG	0.88	0.73–1.05	0.165	0.86	0.72–1.04	0.112
	Women				0.70	0.58–0.84	<0.001
	G	Reference			Reference		
	A	0.93	0.80–1.08	0.333	0.92	0.79–1.06	0.248
	Women				0.70	0.62–0.80	<0.001

**Table 5:** Stratified analysis of rs74035787 and rs1424019 polymorphisms and the risk of HCM in the validation cohort. Stratification criteria were gender and age (≥50 years old).

	**Predictors**	**Women**			**Men**		
		**Odds Ratio**	**CI**	***p*-Value**	**Odds Ratio**	**CI**	***p*-Value**
**rs74035787**	GA	0.20	0.06–0.52	0.003	0.56	0.27–1.14	0.115
	A	0.20	0.06–0.53	0.003	0.57	0.27–1.14	0.118
**rs1424019**	GG	1.03	0.77–1.39	0.830	1.25	0.99–1.58	0.063
	G	1.02	0.80–1.29	0.896	1.14	0.94–1.37	0.173
	**Predictors**	**≥50 yo**			**<50 yo**		
		**Odds Ratio**	**CI**	***p*** **-Value**	**Odds Ratio**	**CI**	***p*** **-Value**
**rs74035787**	GA	0.33	0.16–0.63	0.001	0.70	0.21–2.36	0.559
	Women	0.87	0.71–1.08	0.0208	0.39	0.25–0.60	<0.001
	A	0.33	0.16–0.63	0.001	0.71	0.21–2.35	0.563
	Women	0.87	0.75–1.01	0.073	0.39	0.29–0.53	<0.001
**rs1424019**	GG	1.24	1.01–1.53	0.045	0.92	0.61–1.38	0.686
	Women	0.86	0.70–1.06	0.162	0.39	0.25–0.60	<0.001
	G	1.14	0.96–1.35	0.129	0.93	0.68–1.28	0.674
	Women	0.87	0.75–1.01	0.062	0.39	0.29–0.53	<0.001

**Table 6:** Haplotype frequencies between rs74035787 and rs1424019 polymorphisms in the validation cohort.

Polymorphism		Haplotype Frequency				
rs74035787 G>A	rs1424019 A>G	Patients N = 962	Controls N = 923	*p*-Value	OR	95%CI
**G**	**G**	1441 (0.749)	1341 (0.726)	0.1160	1.13	0.97–1.30
**G**	**A**	466 (0.242)	464 (0.251)	0.5147	0.95	0.82–1.10
**A**	**G**	4 (0.002)	19 (0.010)	0.0035	0.20	0.07–0.59
**A**	**A**	13 (0.007)	22 (0.012)	0.1031	0.56	0.28–1.12

## Data Availability

Further data is available by mailing to the corresponding author (juan.gomezde@sespa.es).

## Supplementary Materials

The following supporting information can be downloaded at: https://www.mdpi.com/article/10.3390/life12060818/s1, Supplementary file with Methods: 1. *DNA Extraction*; 2. *Next Generation Sequencing*; 3. *PCR-RFLP*. Figure S1: *NcoI* digestion and electrophoresis size-fractioning (RFLP) on 4% agarose gel of the rs74035787 G>A in the validation cohort. In addition to the polymorphic site, the rs74035787 PCR-fragment contained two *NcoI* restriction sites that were visualized as constant bands in agarose gels. PCR size: 584 bp; alleles size: G (274 + 222 + 88 bp) and A (496 + 88 bp). M: DNA marker (1 kb DNA ladder), Figure S2: *FENDRR* polymorphism identified by next generation sequencing, Figure S3: Reported frequencies of the rs74035787 and rs1424019 polymorphisms in populations worldwide. Data accessed at the ensembl web (www.ensembl.org). Afr: Africans, Amr: Americans, EAS: East-Asians, EUR: Europeans, SAS: South-Asians; CEU: Utah residents of European ancestry, FIN: Finns, GBR: Great Britain Caucasians, IBS Spanish, TSI: Toscany. Figure S4: FENDRR cellular localization and expression. Bars representing CN-RCI values for the *FENDRR* gene across all cell lines. Expression values (FPKMs) for the *FENDRR* gene are shown for cytoplasm (on top of the bar) and nucleus (on the bottom) compartments. Bars are colored by their absolute nuclear expression. Data accessed at the lncATLAS (crg.eu), Table S1: Genes included in the NGS. Refs. [2,21,39] are cited in the Supplementary File.
