# Supplementary material for: Association of the Genetic Variation in the Long Non-Coding RNA FENDRR with the Risk of Developing Hypertrophic Cardiomyopathy
Source: Life (Basel). 2022 May 30;12(6):818. doi: 10.3390/life12060818 (PMC9225451; doi:10.3390/life12060818)
Supplement: Supplementary file 1 [file life-12-00818-s001.zip › life-1690311-supplementary.pdf]

Supplementary File

Methods

1. DNA extraction

Blood samples were collected in 9 mL tubes with EDTA. DNA from peripheral blood leukocytes was obtained following a salting-out method [1].

2. Next Generation sequencing

All the patients and healthy controls were sequenced for *H19, KCNQ1OT1, MHRT, CARMEN, FENDRR, TINCR, ANRIL, MIAT, PVT1, MALAT1* genes by NGS with a custom AmpliSeq gene panel and Ion Torrent *GeneStudio S5 Plus Sequencer* semiconductor chip technology (Thermo Fisher Scientific, Waltham, MA, EEUU). AmpliSeq panel was designated online (Ion AmpliSeq Designer v4; https://www.ampliseq.com) to cover the coding exons plus at least 5 intron flanking nucleotides of the 10 genes. We compared several primer design options and ordered the one that gave the maximum target sequence coverage. Primer pairs to amplify a total of 316 fragments that covered 98.2% of the target sequence were provided by the manufacturer in only 2 tubes (**Supplementary Table S1**).

The NGS procedure was previously validated [2,3]. Briefly, DNA from each patient and controls was obtained and adjusted to a final concentration of 20 ng/µL. DNA pools containing 100ng of the corresponding DNAs were prepared by pre-quantification the samples with the Qubit 2.0 Fluorometer. The number of samples used to create the pools was decided taking into account the load capacity of the chip, the total length of the target sequences, the dilution of a unique rare allele inside the pool, and the number of reads per amplicon necessary to achieve a minimum coverage of 50×. Each pool was amplified with the Ion AmpliSeq Library Plus Kit in conjunction with Ion AmpliSeq Custom Primer Pool protocols according to the manufacturer's procedures (Thermo Fisher Scientific, Waltham, MA, EEUU) and following the next steps: polymerase chain reaction (PCR) in 2 tubes, partial digestion of the primers with FuPa Reagent, ligation of the bar code adapters, purification by Agencourt AMPure XP Reagent, quantification of the sample (7500 Real Time PCR System), and dilution of the sample to a final concentration of 35 pmol/L.

After amplification, emulsion PCR and enrichment were performed using Ion Chef instrument following the manufacturer's instructions (Thermo Fisher Scientific). The sequencing was performed in the Ion Torrent *GeneStudio S5 Plus Sequencer*, with 540 semiconductor chips. We used a 550-flow runs, which support a template read length of ≈200 bp. The raw GeneStudio S5 Plus Sequencer data were processed with the Torrent Suite v5 software (Thermo Fisher Scientific) to generate sequence reads filtered by the pipeline software quality controls. Reads assembling and variant identification were performed using Variant Caller v5 software and Ion Reporter v5 and and HD Genome One (DREAMgenics S.L., Oviedo, Asturias, Spain) softwares respectively, using FastQ files containing sequence reads and the Ion AmpliSeq Designer BED file software to map the amplicons. We used the Integrative Genome Viewer (Broad Institute, Cambridge, MA, EEUU) for the analysis of depth coverage, sequence quality, and variants identification (**Supplementary Figure S2**). Variants were identified with the somatic sample default algorithm. The raw data were aligned against the reference GRCh38 sequence.

3. PCR-RFLP

We searched online (www.ensembl.org) for *FENDRR* single-nucleotide polymorphisms (SNPs) in high linkage disequilibrium (LD) with the three *FENDRR* polymorphisms identified by NGS.

Therefore, we selected rs74035787 A/G polymorphism for perform a restriction enzyme digestion of a PCR fragment (PCR-RFLP) procedure for genotyping. The information about this variant (flanking sequence, population frequencies and linkage disequilibrium (LD) values) was obtained from the *Ensembl* web site (www.ensembl.org). All the patients and healthy controls were genotyped by polymerase chain reaction (PCR) amplification of genomic DNA with specific primer-pairs (Forward primer: 5' CAAGCAGAATTCCTCCATCCCAGG 3'; Reverse primer: 5' CTCAGAAGCGGGTTATAGGCCCTT 3'; PCR size: 584 bp) followed by digestion with a restriction enzyme *NcoI* (EURx, Gdansk, Pomorskie, Poland) and electrophoresis size-fractioning (RFLP) on 4% agarose gel of the corresponding fragment-alleles. The rs74035787 PCR-fragment contained two *NcoI* restriction sites (C^CATGC/GCATG^G) that were visualized as constant bands in agarose gels (**Supplementary Figure S1**). The PCR Mix contained 19μl Master Mix (dNTPs, H_2O, $MgCl_2$ and reaction buffers), 0.5 μl of Taq polimerase, 0.5 μl of each primer and 3 μl of DNA. The PCR amplification used the following parameters: an initial denaturation at 95°C for 5 min, DNA was amplified for 32 cycles of 95°C for 30 s, 64°C for 1 min, and 72°C for 1 min; and a final extension at 72°C for 5 min. To detect the fragment-alleles, 15 μl of PCR products was digested with 0.3 μl of *NcoI* (10 U μl^{-1}) in 1.5 μl of 10× buffer and 8.2 μl of ddH_2O for 5-6 h at 37°C.

To confirm the reproducibility of the genotyping procedure, in all the assays (PCRs + RFLPs), we included several samples of known genotypes and the genotypes were confirmed in all these control samples. Size of the PCR and allele fragments are summarized in **Supplementary Figure S1.**

Supplementary Table S1. Genes included in the NGS.

Gene/ Gene region	Chr	Num. amplicons	Total Bases	Covered Bases	Missed Bases	Overall Coverage (%)
H19_Promotor	chr11	6	719	714	5	99.3
rs775537394	chr11	1	53	53	0	100
rs878854349	chr11	1	29	29	0	100
rs397508090	chr11	1	8	8	0	100
rs397508091	chr11	1	35	35	0	100
rs397508093	chr11	1	37	37	0	100
rs397508092	chr11	1	29	29	0	100
rs231358	chr11	1	26	26	0	100
KCNQ1OT1_Promotor_Ex1	chr11	14	2219	2145	74	96.7
MALAT1_Promotor	chr11	6	719	719	0	100
H19	chr11	19	2657	2460	197	92.6
MALAT1	chr11	50	8761	8590	171	98.0
MHRT	chr14	10	936	936	0	100
FENDRR	chr16	33	5636	5399	237	95.8
TINCR_Promotor	chr19	5	719	577	142	80.3
TINCR	chr19	18	3763	3045	718	80.9
MIAT_Promotor	chr22	6	719	719	0	100
MIAT	chr22	59	10233	10233	0	100
CARMN_Promotor	chr5	7	839	839	0	100
CARMN	chr5	11	1063	1063	0	100
PVT1_Promotor	chr8	3	719	255	464	35.5
PVT1	chr8	14	2030	1677	353	82.6
ANRIL_Promotor	chr9	5	719	716	3	99.6

ANRIL	chr9	34	4418	4093	325	92.6

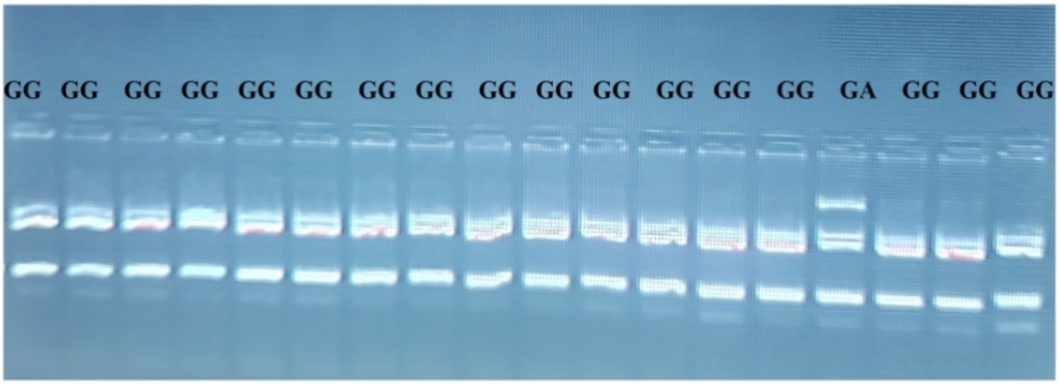

Supplementary Figure S1. *NcoI* digestion and electrophoresis size-fractioning (RFLP) on 4% agarose gel of the rs74035787 G>A in the validation cohort. In addition to the polymorphic site, the rs74035787 PCR-fragment contained two *NcoI* restriction sites that were visualized as constant bands in agarose gels. PCR size: 584 bp; alleles size: G (274 + 222 + 88 bp) and A (496 + 88 bp). M: DNA marker (1 kb DNA ladder).

rs39527 A>G (chr16:86489621)

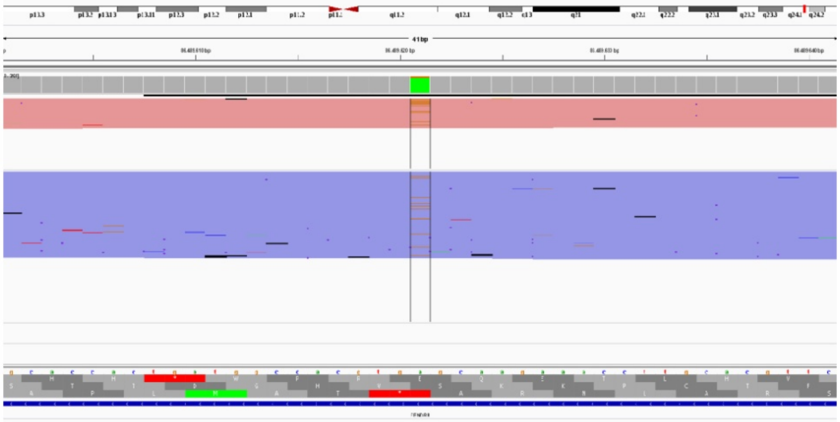

rs39529 G>C (chr16:86488844)

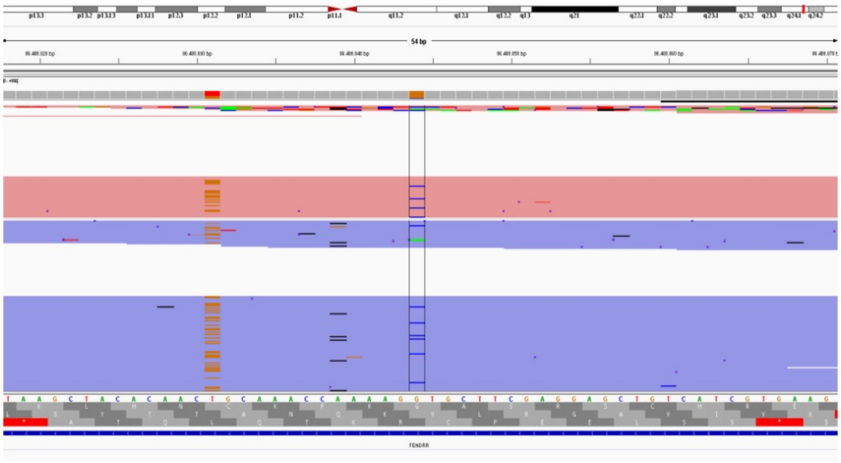

rs40384 T>C (chr16:86490231)

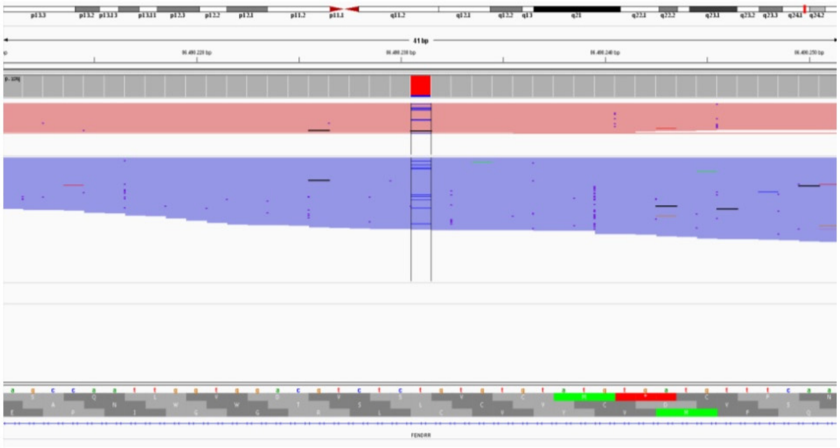

Supplementary Figure S2. *FENDRR* polymorphism identified by next generation sequencing.

rs74035787 G>A

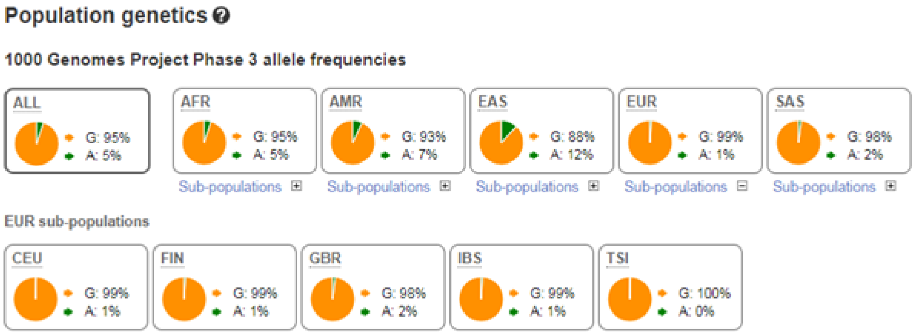

rs1424019 A>G

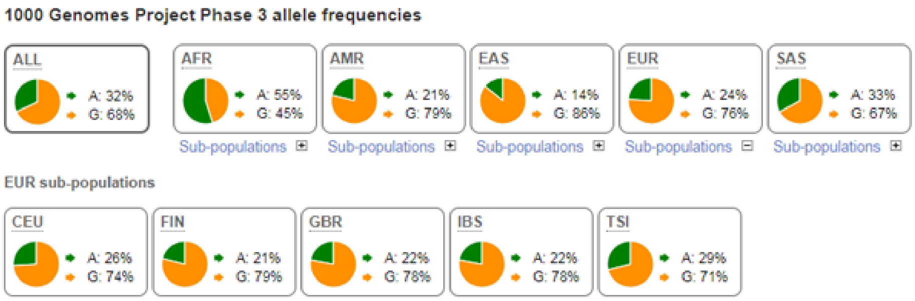

Supplementary Figure S3. Reported frequencies of the rs74035787 and rs1424019 polymorphisms in populations worldwide. Data accessed at the ensembl web (www.ensembl.org). Afr: Africans, Amr: Americans, EAS: East-Asians, EUR: Europeans, SAS: South-Asians; CEU: Utah residents of European ancestry, FIN: Finns, GBR: Great Britain Caucasians, IBS Spanish, TSI: Toscany.

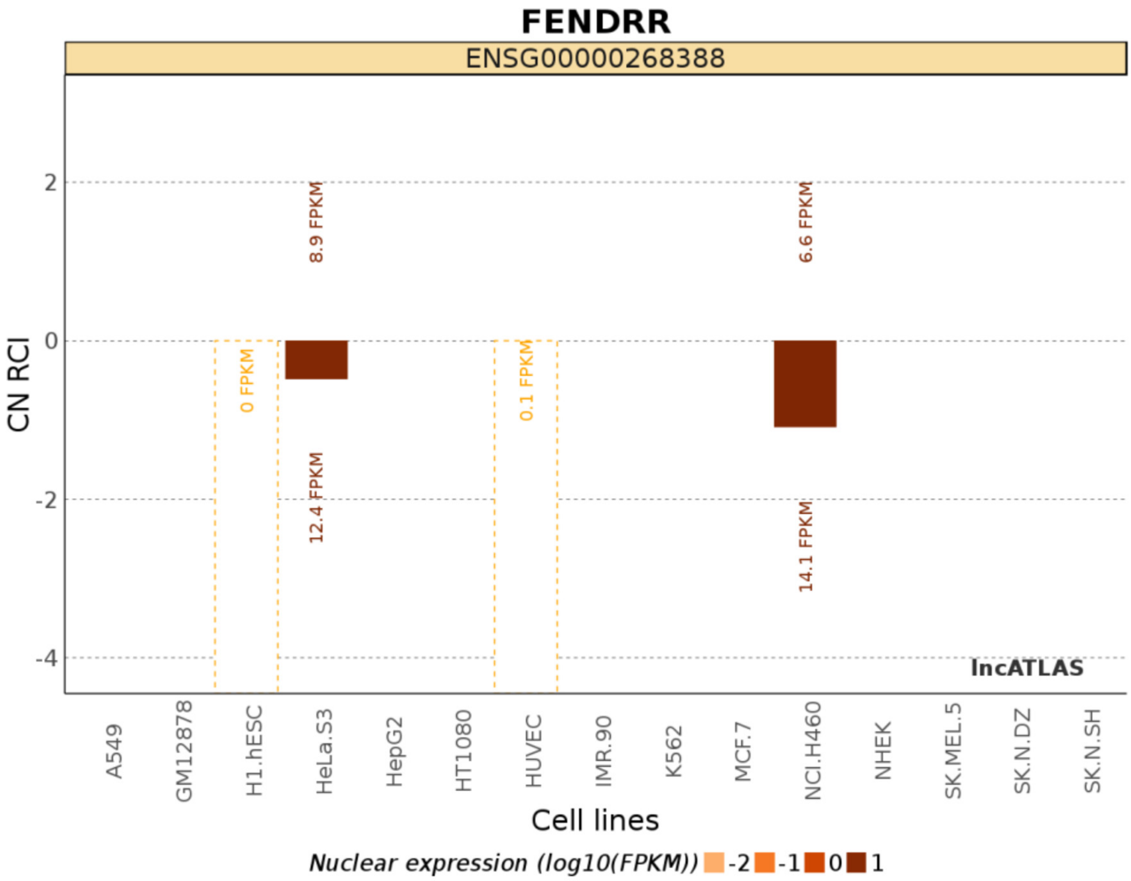

Supplementary Figure S4. *FENDRR* cellular localization and expression. Bars representing CN-RCI values for the *FENDRR* gene across all cell lines. Expression values (FPKMs) for the *FENDRR* gene are show for cytoplasm (on *top* of the bar) and nucleus (on the *bottom*) compartments. Bars are colored by their absolute nuclear expression. Data accessed at the lncATLAS (crg.eu).

References

1. Miller SA, Dykes DD, Polesky HF. A simple salting out procedure for extracting DNA from human nucleated cells. Nucleic Acids Res. 1988;16(3):1215. doi:10.1093/nar/16.3.1215
2. Gómez J, Lorca R, Reguero JR, et al. Screening of the Filamin C Gene in a Large Cohort of Hypertrophic Cardiomyopathy Patients. Circ Cardiovasc Genet. 2017;10(2):e001584. doi:10.1161/CIRCGENETICS.116.001584.
3. Gómez J, Reguero JR, Morís C, et al. Mutation analysis of the main hypertrophic cardiomyopathy genes using multiplex amplification and semiconductor next-generation sequencing. Circ J. 2014;78(12):2963-2971. doi:10.1253/circj.cj-14-0628.
